# Endometrial Cancer in Brazil: Preparing for the Rising Incidence

**DOI:** 10.1055/s-0038-1673644

**Published:** 2018-10

**Authors:** Eduardo Paulino, Angélica Nogueira-Rodrigues, Paul E. Goss, Lilian Faroni, Gustavo Guitmann, Kathrin Strasser-Weippl, Alexandra Bukowski

**Affiliations:** 1 Global Cancer Institute, Boston, Masachussets, United States; 2 Americas Centro de Oncologia Integrado, Rio de Janeiro, RJ, Brazil; 3 Instituto Nacional de Câncer, Rio de Janeiro, RJ, Brazil; 4 Universidade Federal de Minas Gerais, Belo Horizonte, MG, Brazil; 5 Wilhelminen Hospital, Vienna, Austria

Brazil has a population of over 206 million people.^1^ It is estimated that there will be 6.22 new cases of endometrial cancer (EC) for every 100,000 Brazilian women in 2018.^2^ From the estimated 6,000 cases in 2016, GLOBOCAN predicts an increase to 9,372 new cases in 2025 and to 11,963 in 2035.^3^

Obesity and overweight are well recognized, important risk factors for EC.^4^ ^5^ ^6^ ^7^ Data from the Brazilian Institute of Geography and Statistics (IBGE, in the Portuguese acronym) show that, in the past 5 decades, the percentage of women > 20 years old who are overweight or obese increased from 29 to 48% and from 8 to 17%, respectively.^8^ ^9^ ^10^ These trends will only worsen over time, as the IBGE predicts that 38.2% of Brazilian women will be obese by 2022.

Older age is another important risk factor, with ∼ 75% of the patients with type I EC (mainly endometrioid histology) being postmenopausal.^11^ According to the IBGE, the estimated life expectancy in Brazil increased by almost 5 years in the past 15 years, from 73.9 in 2000 to 78.8 years in 2014,^12^ with an estimated life expectancy of more than 80 years by 2023.^13^ ^14^

Surgery for EC should be performed by dedicated gynecologic oncologists^15^ because oncological outcomes improve when EC surgical procedures are performed by high-volume surgeons and in high-volume centers.^16^ However, other than in the USA, in the UK or in Germany, where gynecologic oncology is a subspecialty of its own that requires 3 + years of formal training, there is no society of gynecologic oncology in Brazil, and subspecialty training of 1 to 2 years is offered in only a few institutions. In addition, > 70% of the Brazilian population are covered by the public health system^17^ and have difficulty accessing specialized institutions. Therefore, typically, general gynecologists or surgeons perform gyneco-oncologic surgery.

Two major randomized trials comparing minimally invasive surgery (MIS) through laparoscopy to traditional open hysterectomy^18^ ^19^ for the treatment of EC showed the benefits of MIS in terms of morbidity without compromising the oncologic outcomes. Despite a lack of formal data, based on other studies in low and middle-income countries (LMICs),^20^ it is reasonable to deduct that Brazilian public hospitals lack laparoscopic devices and the capacity to treat all eligible EC patients in the public healthcare system with MIS. Apart from the lack of infrastructure, the availability of MIS also depends on the medical training of the surgeon, which is often insufficient, as discussed above.

Another minimally invasive procedure is robotic surgery. The US Food and Drug Administration (FDA) approved robot-assisted management for gynecologic cancer in 2005 and, in 2009, a survey among the members of the US Society of Gynecologic Oncologists (SGO) revealed that 27% of the responders had performed robotic-assisted surgery, and 66% planned to increase their use of it.^21^ The first Brazilian public robotic platform, the da Vinci Xi Surgical System (Strattner, Rio de Janeiro, RJ, Brazil). was implemented in the Brazilian National Cancer Institute (INCA, in the Portuguese acronym) in 2012. As of June 2018, ∼ 39 robotic systems had been installed in Brazil. It is worth emphasizing that only 4 of these 39 robotic platforms were operating in public hospitals.^22^

Most EC cases are diagnosed at an early stage, and international guidelines recommend adjuvant pelvic radiation or brachytherapy, depending on the risk of recurrence.^23^ ^24^ In 2013, the INCA and the Brazilian Society of Radiotherapy (SBR, in the Portuguese acronym) estimated a shortage of 135 machines and ∼ 90,000 patients per year not receiving radiation therapy despite its indication.^25^ Overall, the public healthcare sector covers only 69.5% of the need for radiotherapy in Brazil, and there are some states with coverage of less than 50%.^25^ Based on recommendations of the Brazilian government in 2014 (1 teletherapy machine per 500,000 inhabitants),^26^ ∼ 412 machines would be needed to cover the entire Brazilian population of 206 million. However, as of 2016, only 292 specialized radiotherapy centers were available for the whole country, of which 223 were in the public system.^27^ This deficit translates into lack of treatment and into an overload of the institutions that have radiotherapy machines, resulting in a median delay in the initiation of treatment of 113.4 days (almost 4 months).^25^ In a recent report, it was estimated that 111,432 patients who required radiotherapy in 2016 did not receive this treatment, and that over 5,000 deaths would probably have been prevented in the cases of the most common cancer types if radiotherapy access were universal in the Brazilian Public Health System (SUS, in the Portuguese acronym).^28^ ^29^ There is room for improvement in three major areas when it comes to EC care and control in Brazil:

1. **Increase the number of radiotherapy machines.** In 2012,^27^ the federal government planned to increase its radiotherapy services, adding 80 linear accelerators by 2015 (32 machines to centers with existing radiotherapy services, and 48 to new centers).^30^ As of April 2017, however, the last bulletin from the Ministry of Health showed that only 10 centers have initiated this expansion, only 1 machine has been installed, and 6 centers had subsequently been excluded from the plan.^30^ With a timeframe of ∼ 30 months between the building of the bunker and the beginning of operations of the system, it is urgent to make progress as soon as possible.
2. **Improve training and expertise of gyneco-oncologists.** It is necessary to expand training programs for radio-oncologists and to promote specialized training in gynecologic oncology, specifically in performing MIS. Oncological surgeons in Brazil have expressed willingness to create a gynecologic oncology subspecialty in the country, which would certainly improve clinical readiness to deal with EC cases.
3. **Support prevention strategies.** Recently, the INCA launched national recommendations on healthy nutrition and exercise to educate the population about the risks of a sedentary lifestyle and poor eating habits and their relation to cancer.^31^ Concerted efforts now need to be made to ensure that these measures of primary prevention reach the general public.

The incidence of EC is rising in both high and middle-income countries due to an increase in Westernized lifestyles, to the rates of overweight and obesity, and to a higher life expectancy. Brazil will face a sharp increase in EC cases in the next decade, which will exacerbate the current shortages and barriers in the treatment of EC. It is therefore imperative to implement EC prevention policies, improve radiotherapy resources, and augment equitable patient access to specialized and well-equipped cancer centers with trained professionals.
